# Integrated Analysis Reveals the Characteristics and Effects of SARS-CoV-2 Maternal–Fetal Transmission

**DOI:** 10.3389/fmicb.2022.813187

**Published:** 2022-01-27

**Authors:** Ziliang Huang, Shuting Xia, Shiqiang Mei, Yanzi Wen, Jialiu Liu, Chengzhi Dong, Wenxin Chen, Peijie Yu, Lianghu Qu, Yanmin Luo, Lingling Zheng

**Affiliations:** ^1^MOE Key Laboratory of Gene Function and Regulation, State Key Laboratory for Biocontrol, School of Life Sciences, Sun Yat-sen University, Guangzhou, China; ^2^Department of Obstetrics and Gynecology, The First Affiliated Hospital of Sun Yat-sen University, Guangzhou, China

**Keywords:** COVID-19, SARS-CoV-2, maternal–fetal transmission, placenta, fetal development, integrated analysis

## Abstract

Severe acute respiratory syndrome coronavirus 2 (SARS-CoV-2) infection has caused a pandemic of coronavirus disease 2019 (COVID-19) and is threatening global health. SARS-CoV-2 spreads by air with a transmission rate of up to 15%, but the probability of its maternal–fetal transmission through the placenta is reported to be low at around 3.28%. However, it is still unclear that which tissues and developmental periods hold higher risks and what the underlying molecular mechanisms are. We conducted an integrated analysis of large-scale transcriptome and single-cell sequencing data to investigate the key factors that affect SARS-CoV-2 maternal–fetal transmission as well as the characteristics and effects of them. Our results showed that the abundance of cytomegalovirus (CMV) and Zika virus (ZIKV) infection-associated factors in the placenta were higher than their primarily infected tissues, while the expression levels of SARS-CoV-2 binding receptor angiotensin-converting enzyme II (ACE2) were similar between lung and placenta. By contrast, an important SARS-CoV-2 infection-associated factor, type II transmembrane serine protease (TMPRSS2), was poorly expressed in placenta. Further scRNA-Seq analysis revealed that ACE2 and TMPRSS2 were co-expressed in very few trophoblastic cells. Interestingly, during the embryonic development stages, the abundance of ACE2 and TMPRSS2 was much higher in multiple embryonic tissues than in the placenta. Based on our present analysis, the intestine in 20th week of embryonic development was at a high risk of SARS-CoV-2 infection. Additionally, we found that during the fetal development, ACE2 and TMPRSS2 were enriched in pathogen infection-associated pathways and may involve in the biological processes related to T-cell activation. In conclusion, our present study suggests that though the placenta provides a good physical barrier against SARS-CoV-2 infection for healthy fetal development, multiple embryonic tissues are under risks of the virus infection, which may be adversely affected once infected prenatally. Therefore, it is necessary to enhance maternal care to prevent the potential impact and harm of SARS-CoV-2 maternal–fetal transmission.

## Introduction

Severe acute respiratory syndrome coronavirus 2 (SARS-CoV-2) infection has caused a worldwide outbreak of coronavirus disease 2019 (COVID-19) and posed a serious threat to public health all over the world. Different from general population, pregnant women often develop a special condition of immune tolerance and may be more susceptible to various infections (Aghaeepour et al., 2017). Common pathogens that infect women during pregnancy include *Toxoplasma gondii*, HIV, rubella, cytomegalovirus (CMV), herpes simplex virus (HSV), hepatitis B virus (HBV), and Zika virus (ZIKV). These viruses can transmit vertically from mother to fetus through the placenta and infect certain fetal organs (Schwartz, 2017). For example, ZIKV is highly neurotropic, causing microcephaly and fetal brain damage like severe cerebral atrophy and intracranial calcifications (Alvarado and Schwartz, 2017). CMV has high viral loads in the fetal liver, resulting in liver calcifications and hepatosplenomegaly (Iwasenko et al., 2011; Diogo et al., 2020). Maternal–fetal transmission of other pathogens can also cause a variety of severe complications, such as miscarriages, congenital malformation, fetal growth restriction, and nervous system damage (Coyne and Lazear, 2016). Recent studies show that though most pregnant women infected by COVID-19 present mild to moderate symptoms, they are at increased risk of pregnancy complications, such as miscarriages, preeclampsia, and preterm labor (Narang et al., 2020). This raises curiosity that whether SARS-CoV-2 infection of pregnant women can cause maternal–fetal transmission and what the underlying mechanism and effects are.

In early small-scale studies of pregnancy complicated with COVID-19, no cases of maternal–fetal transmission were observed (Chen et al., 2020; Schwartz, 2020). However, a case report from Renmin Hospital of Wuhan University showed that IgM antibody was elevated in a newborn baby whose mother was diagnosed with COVID-19, suggesting that SARS-CoV-2 may transmit through the placenta (Dong et al., 2020). The first confirmed case of SARS-CoV-2 transplacental transmission from an infected mother to her neonate is reported in France (Vivanti et al., 2020), and then, more cases of maternal–fetal transmission of SARS-CoV-2 in second and third trimester have been reported (Sukhikh et al., 2021). A Study with a small sample size did not reveal risks of maternal–fetal transmission of SARS-CoV-2 in early pregnancy (Halici-Ozturk et al., 2021), but subsequently, congenital SARS-CoV-2 infection of the fetus and placenta in a woman infected in the first trimester of pregnancy was reported (Valdespino-Vazquez et al., 2021). Therefore, maternal–fetal transmission of SARS-CoV-2 is possible throughout the entire pregnancy.

The general steps of virus infecting and crossing the placenta include host receptor/co-receptor recognition, uptake into cells of placenta, and hijacking cellular processes for viral replication (Schneider-Schaulies, 2000). It is reported that the receptors for CMV, rubella, ZIKV and HBV, are highly expressed in the human placental tissues (Vyas et al., 2018; Pique-Regi et al., 2020). Recent studies also suggest that at least two conditions are needed for maternal–fetal transmission of SARS-CoV-2: (a) The virus can reach the placenta; and (b) angiotensin-converting enzyme II (ACE2), known as the recognition receptor of SARS-CoV-2 and key to virus infection (Shang et al., 2020), must be present in the placenta (Gengler et al., 2021). These conditions have been continually proven by various studies, indicating that SARS-CoV-2 has the ability to transmit vertically. However, the maternal–fetal transmission probability of SARS-CoV-2 is reported to be low, around 3.28% (Yuan et al., 2021). Why is the transmission rate relatively low so far? Are there any other factors affecting the risk of the maternal–fetal transmission of SARS-CoV-2?

The successful entry of SARS-CoV-2 into host cells depends not only on the receptor, but also on an important SARS-CoV-2 infection-associated factor, the type II transmembrane serine protease (TMPRSS2), for S protein priming (Bertram et al., 2011; Xiao et al., 2020). Therefore, the expression of both factors should be taken into account simultaneously to consider the risk of SARS-CoV-2 maternal–fetal transmission.

Generally, SARS-CoV-2 mainly spreads through the air, infects into the lung, and causes substantial respiratory pathology as ACE2 is highly expressed in lung alveolar cells (Sungnak et al., 2020). On the other hand, ACE2 expressed in multiple extrapulmonary tissues along with dysregulation of immune responses and the interaction between ACE2 and TMPRSS2 could be decisive for extrapulmonary manifestations and the clinical outcome of COVID-19 (Gupta et al., 2020; Zipeto et al., 2020). However, it seems that the fetal lung is not the primary organ targeted by SARS-CoV-2 when transplacental transmission occurs. The effects of intrauterine SARS-CoV-2 infection on the health of the fetus remain poorly studied. Only few cases reported abnormal conditions including fetal growth restriction, hydropericardium, periventricular leukomalacia, and intraventricular hemorrhage (Sukhikh et al., 2021). So how is the distribution of SARS-CoV-2 infection-associated factors in the placenta and the risk of SARS-CoV-2 infecting specific organ of the fetus like? Is there any correlation between the infection period and the impact on fetal organs?

To answer these questions, we conducted an integrated analysis of large-scale transcriptome sequencing data of multiple tissues in normal people and during fetal development and single-cell sequencing data of the maternal–fetal interface. We aim to investigate the expression levels and changes of ACE2 and TMPRSS2 in different tissues and periods of fetal development and their potential functions, to uncover the characteristics and effects of maternal–fetal transmission of SARS-CoV-2.

## Materials and Methods

### Data Acquisition

The expression matrix of the bulk RNA-Seq data containing FPKM values for 20,050 genes in 27 tissues was downloaded from Fagerberg et al. (2014) supplementary materials. Gene expression data of lung and placenta were taken out for boxplot in R (version 4.0.5) using package ggpubr (version 0.4.0) and ggplot2 (version 3.3.3).

The microarray data of placenta at different stages of pregnancy were downloaded from GEO under the accession number GSE9984 of Mikheev et al. (2008). This study contains the comparison of placental gene expression profiles of three gestational stages [first trimester (45–59 days), second trimester (109–115 days), and C-section term placentae], with four replicates each.

The single-cell RNA-Seq data of maternal–fetal interface are deposited at ArrayExpress, with experiment codes E-MTAB-6701 (Vento-Tormo et al., 2018). The gene expression matrix and the cell type annotation file were downloaded and reanalysis using R (version 4.0.5).

The bulk RNA-Seq data in 6 tissues between the 10th and 20th week of fetal development (Szabo et al., 2015) can be download from NCBI Bioproject database (PRJNA270632). The mean expression of each tissues’ replicates was taken out for downstream analysis.

### Data Processing and Analysis

#### Single-Cell RNA-Seq Data Analysis

The gene expression matrix contains unique molecular identifier (UMI) count was converted to Seurat object using R package Seurat (version 4.0.3). Cells with fewer than 500 detected genes and for which the total mitochondrial gene expression exceeded 20% were removed and mitochondrial genes and genes that were expressed in fewer than three cells were also removed by Roser et al. We used sctransform for the normalization with default parameters (Hafemeister and Satija, 2019). Then perform dimensionality reduction by PCA and UMAP embedding using function RunPCA, RunUMAP, FindNeighbors, and FindClusters with dims = 1:30. We annotated the cell types using the annotation files adopted from the publication of Roser et al. The UMAP plot was generated using function DimPlot, and the dot plot of the expression level of ACE2, and TMPRSS2 was generated using function DotPlot and modified using ggplot2 (version 3.3.3).

#### Fetal Development Analysis

To get the risk assessment criterion, the adult lung gene expression was taken from the 27 different adult tissues data and then merge with the gene expression of 6 fetal tissues data between 10th and 20th week of fetal development. In order to make the tissues comparable, we applied the quantile normalization (Bolstad et al., 2003) on the merge matrix which ensure the distribution of gene expression intensities for each samples the same, using function normalize quantiles of the R package preprocessCore (version 3.3.3). We used the expression values of ACE2 and TMPRSS2 in placental and adult lung tissue as baseline. If the expression values of both risk factors in an organ were above the maximum value of the baseline threshold range, then we set the organ as High risk; if only one risk factor was above the maximum value of the baseline threshold range, then we classified the organ as Medium–high risk; if the expression values of both risk factors in an organ were within the baseline threshold range, then the organ was classified as Medium risk; If only one risk factor is within the baseline threshold range, the organ is classified as Medium-low risk; if both risk factors are below the minimum value of the baseline threshold range, the organ is classified as low risk.

#### WGCNA Analysis and Enrichment Analysis

The R package WGCNA (version 1.70) was used to explore gene networks of fetal development gene expression data. Co-expressed genes were clustered into networks and created “modules,” which are defined as groups of highly interconnected genes. We use a soft threshold power of 12 for transforming the unsigned co-expression matrix based on the correlation between genes into an adjacent matrix. After module identification, phenotypic data (which tissue and development stage come from) were correlated with modules. We analyzed the correlation between eigengene with all genes in the brown module and the correlation between all genes in the module with the intestine tissue. Then, genes with high correlation coefficients were selected as hub genes. Gene Significance (GS) or Module Membership (MM) were calculated by correlating the gene’s expression with the respective phenotypic trait or the respective module’s expression, respectively.

High relationship genes (genes with MM > 0.79 and GS > 0.24) in intestine vs. brown module were used for enrichment analysis using the R package clusterProfiler (version 3.18.1). Gene Ontology (GO) enrichment analysis was performed using the function enrichGO, and Kyoto Encyclopedia of Genes and Genomes (KEGG) enrichment analysis was performed using the function enrichKEGG.

## Results

### SARS-CoV-2 Infection-Associated Factors Are Expressed Unevenly in the Placenta

**Figure 1:**
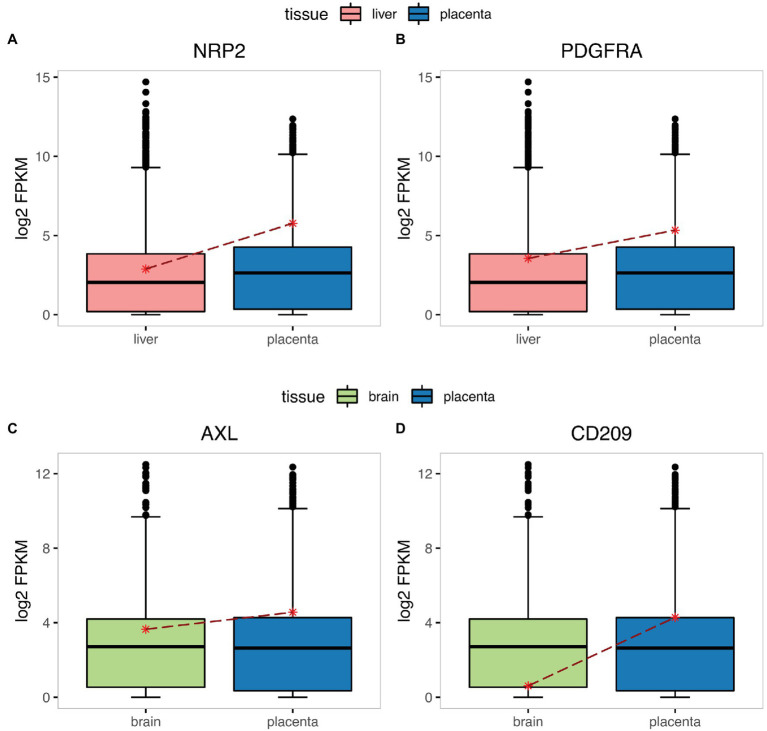
The expression of CMV and ZIKV infection-associated factors in targeted tissues and placenta. The boxplot represents the expression level of CMV infection-associated factors NRP2 **(A)**, PDGFRA **(B)** in the liver and placenta, and ZIKV infection-associated factors AXL **(C)**, and CD209 **(D)** in the brain and placenta, the asterisk marks the mean expression level of genes. The y-axis indicates the normalized expression (log2-transformed FPKM + 1) of genes. FPKM, Fragments Per Kilobase of exon model per Million mapped fragments.

**Figure 2:**
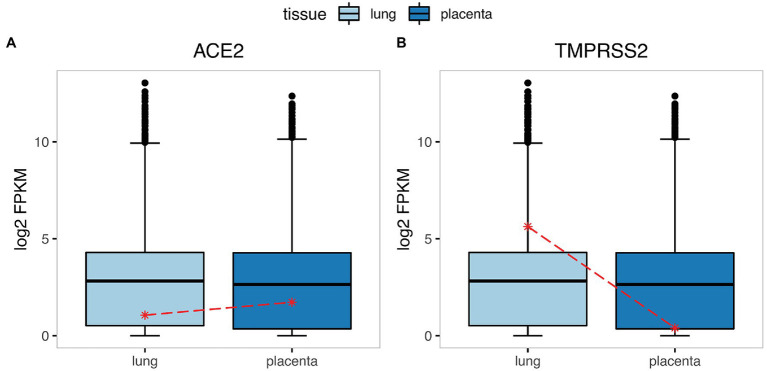
The expression of the SARS-CoV-2 infection-associated factors in the lung and placenta. The boxplot represents the expression level of ACE2 **(A)** and TMPRSS2 **(B)** in the lung and placenta, the asterisk marks the mean expression level of genes. The y-axis indicates the normalized expression (log2-transformed FPKM + 1) of genes.

As CMV and ZIKV were commonly studied that can be transmitted vertically and cause fetal complications, such as microcephaly, multiorgan disease, congenital malformations, and intrauterine growth restriction (Schwartz, 2017), so firstly, we examined the expression levels of several CMV infection-associated factors (NRP2, PDGFRA) and ZIKV infection-associated factors (AXL, CD209) to investigate whether there is correlation between the expression levels of infection-associated factors and the ability of viruses infecting the placenta and fetus. Remarkably, we found that the expression levels of these factors were all higher in the placenta than the primary targeted tissues infected by the corresponding virus (liver for CMV and brain for ZIKV; Figure 1), consistent with the maternal–fetal transmission characteristics of two viruses. Then the expression levels of certain SARS-CoV-2 infection-associated factors (ACE2, TMPRSS2) were explored. The result showed that although ACE2 expressed comparable in placenta and lung, the abundance of TMPRSS2 was relatively high in lung but was almost absent in placenta (Figures 2A,B). In addition, we analyzed the expression level of these two genes in placenta at different stages of pregnancy. We found that ACE2 was not significantly changed during early, middle, and late pregnancy (Supplementary Figure S1A), and consistently, TMPRSS2 was hardly detected in the placenta during the whole pregnancy (Supplementary Figure S1B). The uneven distribution of the two infection factors in the placenta may limit the infection of SARS-CoV-2 into the placenta and its further vertically spread. We therefore wanted to investigate which cell types in the placenta simultaneously express these two factors.

### ACE2 and TMPRSS2 Are Co-expressed in Very Few Trophoblastic Cells

**Figure 3:**
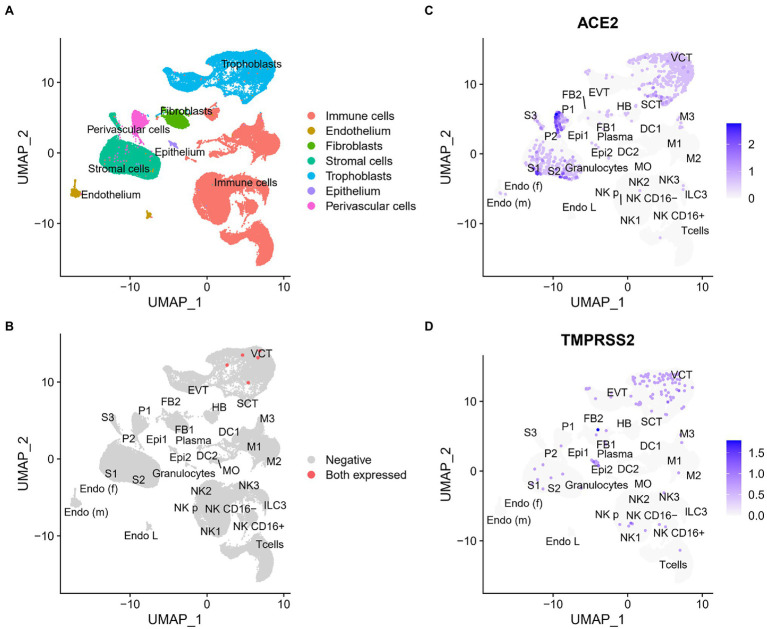
Identification and expression of cell types at the maternal–fetal interface. **(A)** The UMAP clusters plot of cell types at the maternal–fetal interface using scRNA-seq analysis, dots means a single cell and colors indicate cell type {epithelium (Epi1, Epi2), endothelium [Endo L, Endo (f), Endo (m)], stromal cells (S1, S2, S3), perivascular cells (P1, P2), fibroblasts (FB1, FB2), trophoblast (VCT, SCT, EVT), and immune cells (HB, DC1, DC2, MO, NK CD16−, NK CD16+, T cells, granulocytes, M1, M2, M3, NKp, NK1, NK2, NK3, ILC3, and plasma)}. DC, dendritic cells; M, macrophages; MO, monocytes; S, stromal cells; Endo, endothelial cells; Epi, epithelial glandular cells; FB, fibroblasts; HB, Hofbauer cells; P, perivascular cells; ILC, innate lymphocyte cells; SCT, syncytiotrophoblast; VCT, villous cytotrophoblast; EVT, extravillous trophoblast. And the points in **(B)** colored red are the cells co-expressed ACE2 and TMPRSS2. UMAP visualization of the log-transformed, normalized expression of ACE2 **(C)**, and TMPRSS2 **(D)** at the maternal–fetal interface using scRNA-seq analysis, dots mean a single cell and colors indicate different expression level.

Further, we investigated the cell types in the placenta which are possibly vulnerable to be infected by SARS-CoV-2 utilizing the single-cell sequencing (scRNA-Seq) data of maternal–fetal interface (Vento-Tormo et al., 2018). Firstly, cell types were annotated according to the result by Vento-Tormo et al., and 32 types of cells were identified and merged into main clusters (Figure 3A), including three types of trophoblasts, three types of endothelia, three types of stromal cells, two types of epithelia, two types of perivascular cells, two types of fibroblasts, and 17 types of immune cells (details in Section “Materials and Methods”).

We then investigated the distribution of ACE2 and TMPRSS2 in different cell types (Figures 3C,D). Similar to the findings of bulk RNA-seq data, the proportion of ACE2+ and TMPRESS2+ cells were low in many cell types of the placenta. In 32 types of cells, 19 and 17 cell types contained ACE2+ cells and TMPRESS2+ cells, respectively. ACE2 was mainly expressed in perivascular cells, stromal cells, trophoblasts, and TMPRESS2 was mainly expressed in epithelium, fibroblasts, and trophoblasts.

We next sought to find out the cell types in which the two cofactors were expressed simultaneously. The results (Figure 3B) showed that most placental cells did not express ACE2 and TMPRSS2 at the same time, and they were co-expressed in only five cells which all belonged to villous cytotrophoblasts (VCT), which created very unfavorable conditions for SARS-CoV-2 to infect the placenta. Results taken together indicated that the placenta provides a potent barrier against SARS-CoV-2 infection, but what is the situation like within the embryos?

### SARS-CoV-2 Infection-Associated Factors Are Highly Expressed in Multiple Embryonic Tissues During Development

**Figure 4:**
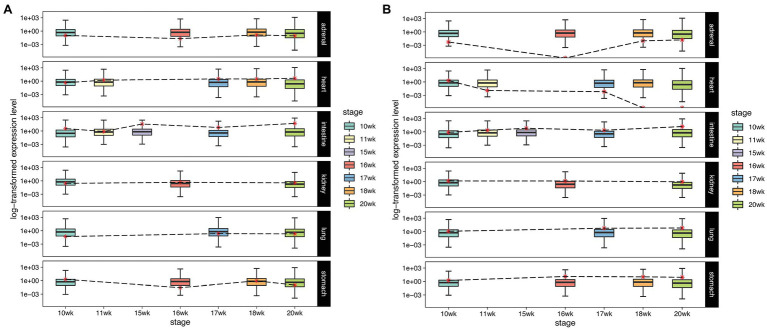
The expression level of SARS-CoV-2 infection-associated factors. The gene expression level in multiple embryonic tissues, the red dots and dotted lines represent the expression level of ACE2 **(A)** and TMPRSS2 **(B)** in different stages. The y-axis indicates the normalized expression (log10-transformed FPKM) of genes.

The expression levels of ACE2 and TMPRSS2 in 6 tissues (adrenal gland, heart, intestine, kidney, lung, and stomach) between 10th and 20th week of fetal development were investigated (Figure 4). We found that compared with the low expression level of ACE2 in placenta, the abundance of ACE2 was higher in multiple fetal tissues (almost near the 50th percentile), especially in the heart, intestine, kidney, and lung, with an average expression level exceeding the median expression level of all genes in the tissue. More interestingly, expression of ACE2 in these tissues gradually increased with the fetal development stages. By the 20th week, the expression levels of ACE2 have exceeded three quarters of all genes in heart and intestine and reached the median expression level in kidney and lung (Figure 4A).

Next, we studied the expression levels and changes of TMPRSS2 in these tissues during embryonic development (Figure 4B). We noticed that the abundance of TMPRSS2 varied among different fetal tissues, but it exceeded the median expression level in intestine, kidney, lung, and stomach and kept at a stable expression level during different stages, which significantly differed from its low abundance in placenta. The results taken together suggest that in multiple fetal tissues between the 10th and 20th week of fetal development, it is sufficient for SARS-CoV-2 to infect successfully.

**Figure 5:**
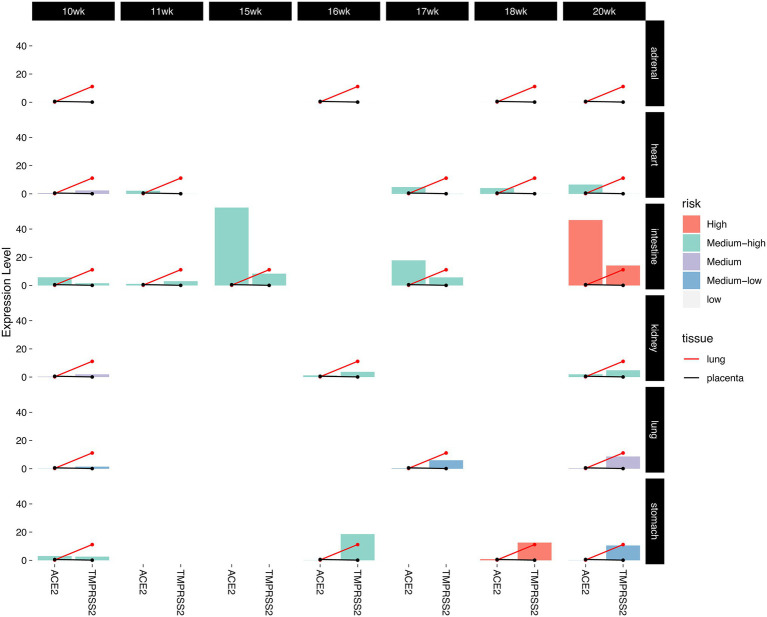
The risk of SARS-CoV-2 infection. The red line is the baseline which represents the expression levels of two SARS-CoV-2 infection-associated factors in adult lung tissue, the black line is the baseline which represents the expression levels of two SARS-CoV-2 infection-associated factors in placenta tissue. The bars reflect the expression levels of two factors in six fetal tissues during fetal development. The colors represent the different risks.

To further study the risk of SARS-CoV-2 infection in different fetal tissues and development stages, we established a risk assessment criterion. The expression levels of ACE2 and TMPRSS2 in adult lung and placental tissue were used as the baseline, and we classified the tissues’ intrusion risks into five levels: high, medium–high, medium, medium–low, and low (see Section “Materials and Methods,” Supplementary Table S1). Our results found that in 6 tissues between 10th and 20th week of fetal development, stomach and intestine tissues showed high risk or medium–high risk in most of the stage (Figure 5). In addition, heart, kidney, and lung were at mediate risk or medium–low risk in various fetal development stages. Adrenal tissue was at low risk from 18th to 20th week. Specifically, we found that ACE2 and TMPRSS2 expression levels were consistently higher in fetal intestinal tissue than the placental baseline expression level. In particular, both factors were higher in the intestine than the lung and placental baseline expression levels by 20th week.

### ACE2 and TMPRSS2 Are Collectively Enriched in Intestinal Pathogen Infection-Related Pathways

**Figure 6:**
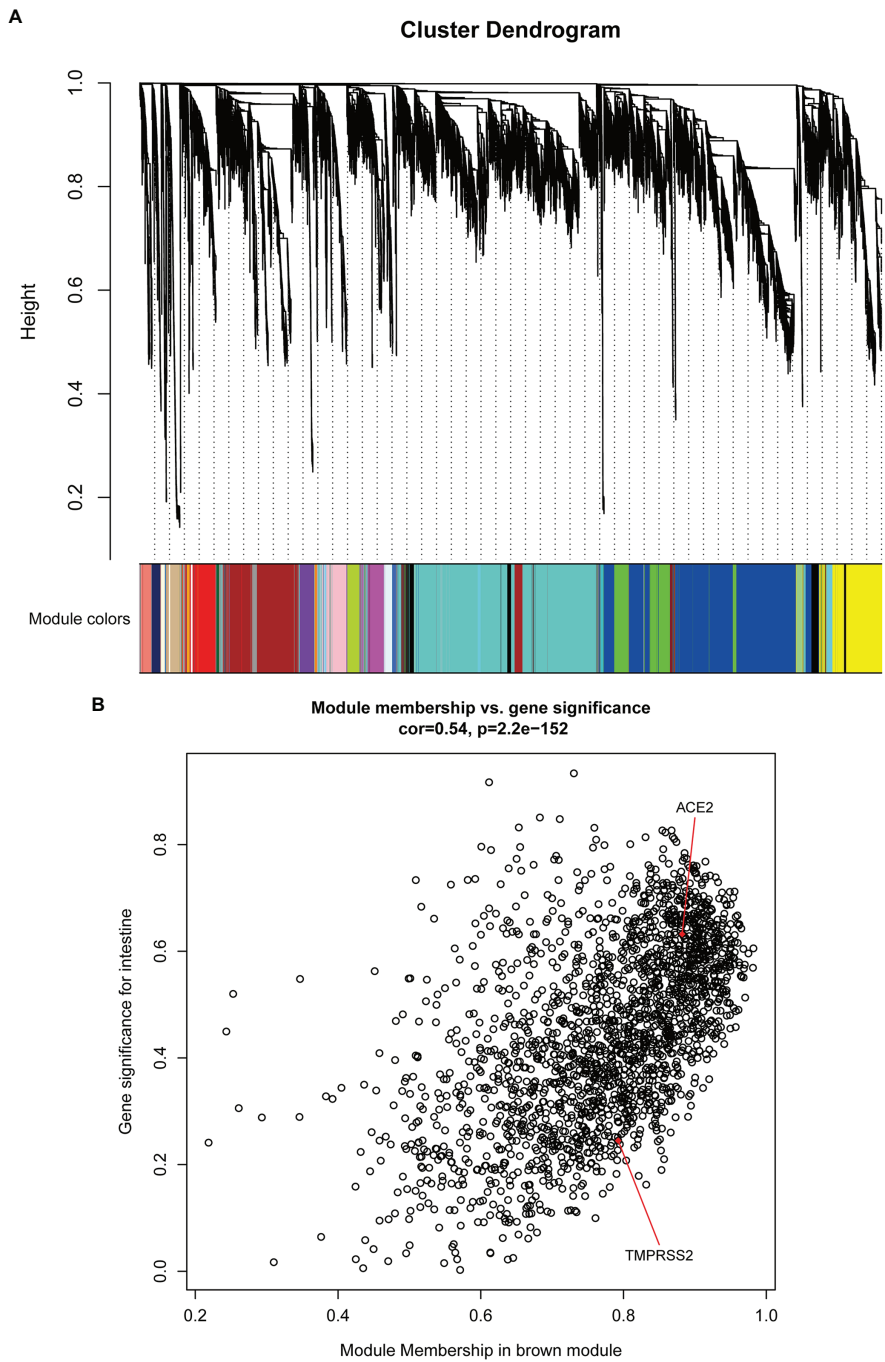
WGCNA analysis of organs of the fetus. **(A)** Gene dendrogram obtained by average linkage hierarchical clustering. The color row underneath the dendrogram shows the module assignment determined by the Dynamic Tree Cut. **(B)** The scatterplot of Gene Significance (GS) for intestine vs. Module Membership (MM) in the brown module. GS is (the absolute value of) the correlation between the gene and the trait, and for each module, MM is the correlation of the module eigengene and the gene expression profile. The red points are ACE2 or TMPRSS2 and have a highly significant correlation between GS and MM in this module.

To look for gene modules that may be significantly related to development stages and specific tissues, we next performed weighted correlation network analysis (WGCNA) on the six fetal tissues during embryonic development stages. Results showed that totally 30 gene modules with similar gene expression patterns were identified (Figure 6A), which were not strongly related to development stages but were significantly related to specific tissues (Supplementary Figure S2). Surprisingly, ACE2 and TMPRSS2 were commonly enriched in the Brown module, which had the most significant correlation with the intestine (Pearson correlation coefficient = 0.59, value of *p* = 0.003). In our previous analysis, the intestine was at a high risk of being infected by SARS-CoV-2 during fetal development, so further we focused on the Brown module and sought to find out the hub gene set of it. It is found that ACE2 and TMPRSS2 were both hub genes in the Brown module, indicating that a group of genes with similar expression patterns as ACE2 and TMPRSS2 may influence the intestine organ development collectively (Figure 6B).

**Figure 7:**
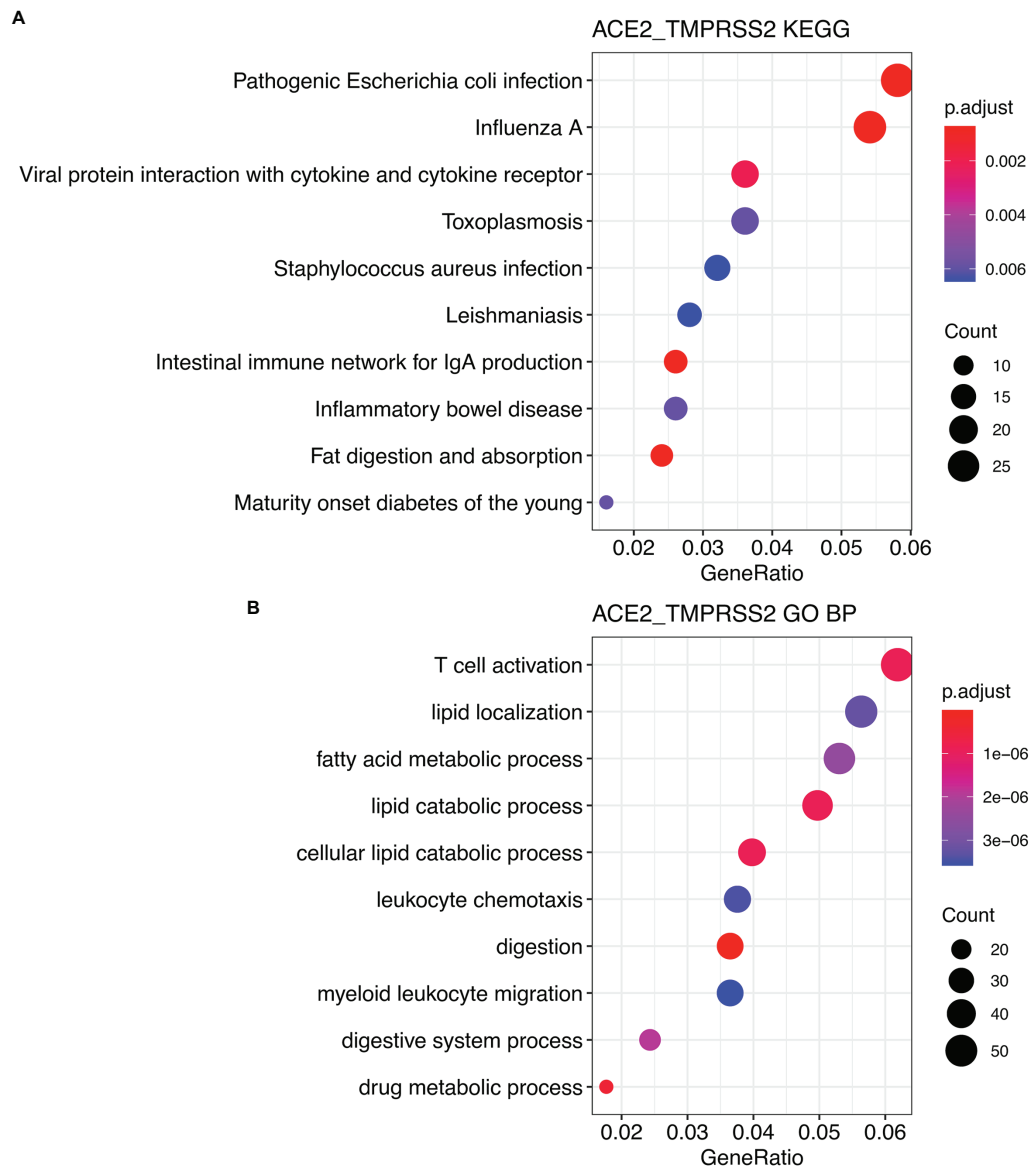
Enrichment analysis of genes of the brown module. Kyoto Encyclopedia of Genes and Genomes pathway **(A)** and biological process analysis results **(B)** of high relationship genes in intestine vs. brown module. One thousand thirty one genes (genes with MM > 0.79 and GS > 0.24) were used for enrichment analysis.

Subsequently, we analyzed the function of hub genes in the Brown module and the biological pathways that they were involved in. These genes were significantly enriched in pathways associated with infection processes, providing further evidence that ACE2 and TMPRSS2 were co-expressed with genes associated with pathogen infection. KEGG analysis also suggested that hub genes may be related to intestinal pathogen infection pathways (Figure 7A), and GO analysis showed that more than 6% of these genes were involved in the biological processes related to T-cell activation (Figure 7B).

## Discussion

Since the outbreak of COVID-19, there have been many contradictions and controversies over whether SARS-CoV-2 can transmit through the placenta (Juan et al., 2020; Kotlyar et al., 2021). There are studies inferring whether maternal–fetal transmission happens (Gengler et al., 2021) or not (Pique-Regi et al., 2020) base on the expression level of ACE2 in placenta. Our present study showed that the expression levels of CMV and ZIKV infection-associated factors were higher in the placenta than their primary infection tissues (liver for CMV and brain for ZIKV), in consistence with previous study results, which can reflect the probability of maternal–fetal transmission to a certain extent. However, the expression levels of ACE2 were similar between lung and placenta in terms of both the absolute value and relative percentile, which cannot fully explain the low maternal–fetal transmission rate in clinical reports. Therefore, we propose that the risk of SARS-CoV-2 maternal–fetal transmission cannot be determined solely by the expression level of ACE2. Then we found that the expression level of an important SARS-CoV-2 infection-associated factor, TMPRSS2, was significantly different between lung and placenta. It was highly expressed in lung while poorly expressed in placenta, which we considered as the reason for the relatively low maternal–fetal transmission rate of SARS-CoV-2.

Further, ACE2 and TMPRSS2 were found to be co-expressed in VCT, a subtype of trophoblasts. Trophoblast is the major cell type of the placenta, and an important part of the maternal–fetal interface, that is the key basis of fetal development (Jansson, 2016). There are three main subtypes: VCT, syncytiotrophoblast (SCT), and extravillous trophoblast (EVT). VCTs are proliferative progenitor cells that play a critical role in various physiological processes in trophoblast differentiation and placenta maturation, including villi formation, nutrient exchange, and hormone production (Kreis et al., 2020). Trophoblast dysfunction is associated with several pregnancy complications, such as placenta accreta, preeclampsia, and fetal growth restriction, and the affected offspring are predisposed to certain health problems in the future (O’Tierney-Ginn and Lash, 2014). Therefore, we consider that though the probability of transplacental transmission of SARS-CoV-2 is not high, it may cause damage to fetal development once transmitted vertically.

Subsequently, our results showed that the abundance of ACE2 and TMPRSS2 was much higher in multiple embryonic tissues during different fetal development stages than in the placenta and gradually increased. In addition, estimated by our risk assessment criterion, stomach, and intestine were at a high or medium–high risk of SARS-CoV-2 infection. The results taken together suggested that SARS-CoV-2 may be transmitted from infected mothers to the fetus during the whole pregnancy and present adverse impact on multiple organs of the fetus, especially the digestive system. Though existing researches showed that most newborns delivered by COVID-19 pregnant women were asymptomatic, few with slight respiratory symptoms (Juan et al., 2020), there were also cases reporting neonatal gastrointestinal symptoms, such as vomiting and abdominal distension (Zhu et al., 2020; Fan et al., 2021) and fetal organ dysfunction including right ventricular hypertrophy, hydropericardium, and intraventricular hemorrhage (Sukhikh et al., 2021). Thus, we call for comprehensively fetal organ screening for pregnancy complicated with COVID-19.

Moreover, we found that ACE2 and TMPRSS2 were commonly enriched in intestinal immunity-related pathways and may involve in the biological processes related to infection and T-cell activation. Similarly, a recent study proposed that during pregnancy, maternally restricted infection can directly impose epigenetic changes on fetal intestinal epithelial stem cells, leading to long-lasting impacts on intestinal immune homeostasis (Lim et al., 2021). Further postnatal monitoring of offspring born to COVID-19 pregnant women is needed to investigate whether there will be long-term effect of SARS-CoV-2 maternal–fetal transmission.

However, as a preliminary study, there are still some limitations in our study. First of all, all analyses are based on transcriptome data of various tissues and cells, which may not truly represent the overall state of protein expression *in vivo*. In addition, the data are derived from physiological conditions of healthy people, but the infection of SARS-CoV-2 may affect the transcriptional profiles of the maternal–fetal interface and fetal tissues. Thus, the results should be interpreted with intense care and further clinical studies and confirmation experiments are necessary to get a more comprehensive conclusion.

At a time when global epidemic of COVID-19 is still serious, we call for enhanced maternal care throughout the whole pregnancy to prevent the potential impact and harm of SARS-CoV-2 maternal–fetal transmission. At the same time, more attention should be paid on relevant research, to explore the molecular mechanism of maternal–fetal transmission of the virus and provide guidance for clinical practice in the health of mothers and fetuses.

## Data Availability Statement

The datasets presented in this study can be found in online repositories. The names of the repository/repositories and accession number(s) can be found in the article/Supplementary Material.

## Author Contributions

LZ designed the research. LZ and YL supervised this study and revised the manuscript. ZH and YW conducted programming. SM, CD, WC, and PY downloaded, managed, and processed the data. JL and SX searched for fetal development-related literatures. LQ provided guidance for the project. ZH and SX drafted the main manuscript text collectively. All authors contributed to the article and approved the submitted version.

## Funding

This research was funded by National Natural Science Foundation of China (31771459 and 82071657); and Guangdong Province (2021A1515010542). This research is supported by Pearl River S&T Nova Program of Guangzhou (201806010151), Guangdong Province Key Laboratory of Computational Science, and the Guangdong Province Computational Science Innovative Research Team (in part).

## Conflict of Interest

The authors declare that the research was conducted in the absence of any commercial or financial relationships that could be construed as a potential conflict of interest.

## Publisher’s Note

All claims expressed in this article are solely those of the authors and do not necessarily represent those of their affiliated organizations, or those of the publisher, the editors and the reviewers. Any product that may be evaluated in this article, or claim that may be made by its manufacturer, is not guaranteed or endorsed by the publisher.
