# Supplementary material for: Integrated Analysis Reveals the Characteristics and Effects of SARS-CoV-2 Maternal–Fetal Transmission
Source: Front Microbiol. 2022 Jan 27;13:813187. doi: 10.3389/fmicb.2022.813187 (PMC8828581; doi:10.3389/fmicb.2022.813187)
Supplement: Supplementary file 1 [file Image_1.PDF]

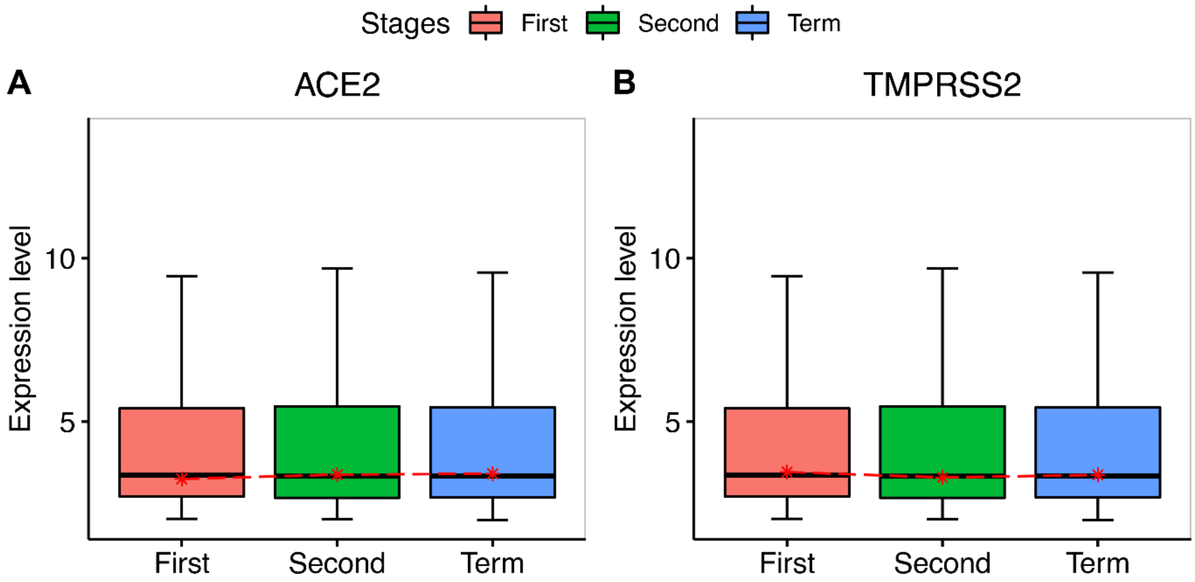

Figure S1. The expression level of the SARS-CoV-2 invading associated factors in the placenta at different stages of pregnancy.

The boxplot represents the expression level of ACE2 (A), and TMPRSS2 (B) in the placenta, the asterisk marks the mean expression level of genes. The X-axis indicates the three gestational stages (First (45-59 days), Second (109-115 days), and Term (C-section term placentae)). The Y-axis indicates the normalized expression level of genes.

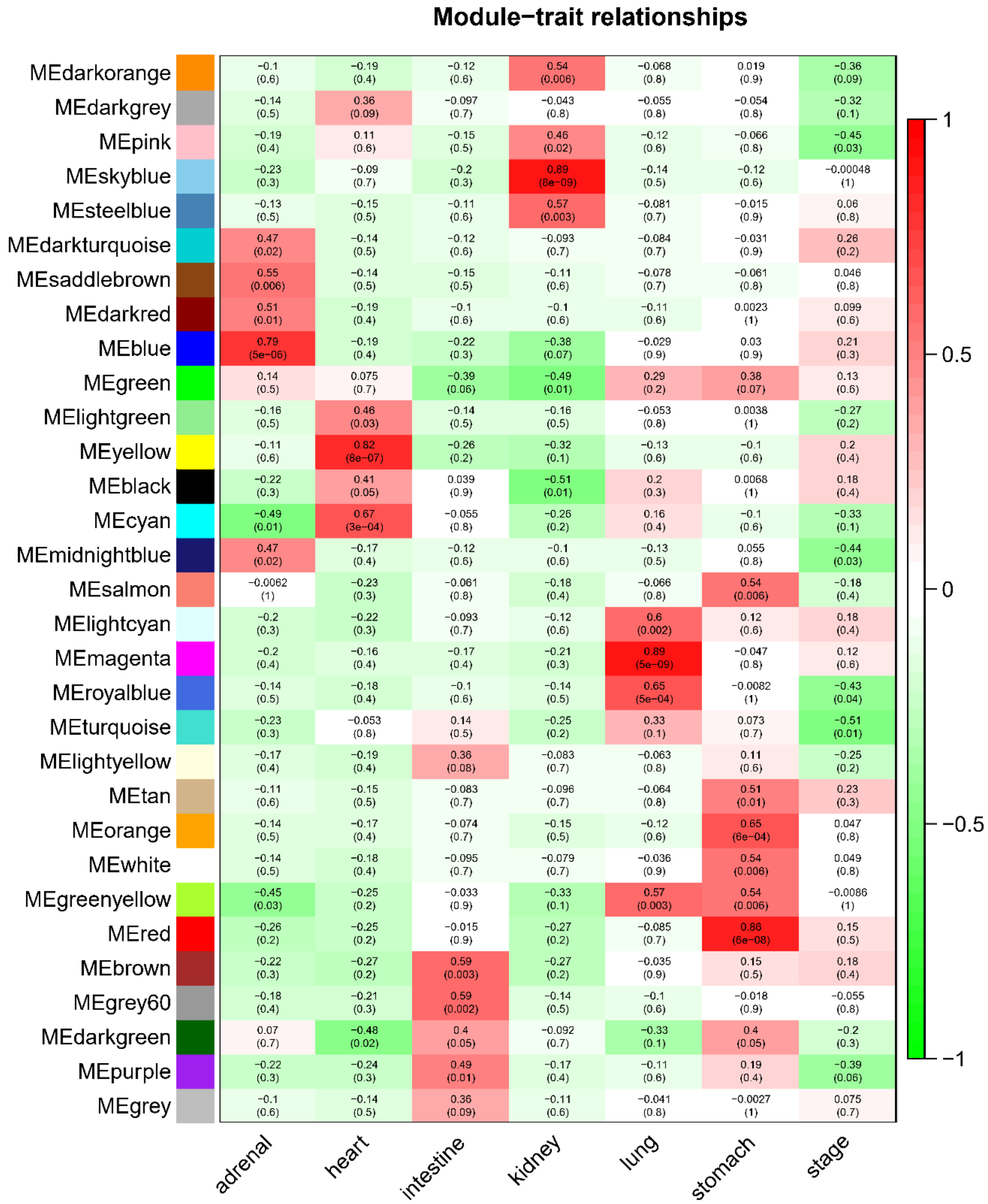

Figure S2. Module-trait associations.
Each row corresponds to a module eigengene, and each column corresponds to a trait parameter. The module eigengene is defined as the first principal component of a given module and considered a representative of the gene expression profiles in a module. Each grid contains the corresponding correlation coefficient and p-value, calculated based on eigengene expression and clinical traits. The grid is color-coded by correlation according to the color bar of the correlation.
