# Supplementary material for: Integrated Analysis Reveals the Characteristics and Effects of SARS-CoV-2 Maternal–Fetal Transmission
Source: Front Microbiol. 2022 Jan 27;13:813187. doi: 10.3389/fmicb.2022.813187 (PMC8828581; doi:10.3389/fmicb.2022.813187)
Supplement: Supplementary file 2 [file Table_1.PDF]

Table S1. The risk of SARS-CoV2 invasion

ACE2	lung_ace2	placenta_ace2	TMPRSS2	lung_tmprss2	placenta_tmprss2	group	organ	stage	risk
46.33404321	0.2458239	0.6059364	14.11181428	11.1761965	0.1297935	intestine_20wk	intestine	20wk	High
0.826824847	0.2458239	0.6059364	12.5298738	11.1761965	0.1297935	stomach_18wk	stomach	18wk	High
2.146764905	0.2458239	0.6059364	0.016688038	11.1761965	0.1297935	heart_11wk	heart	11wk	Medium-high
4.775930917	0.2458239	0.6059364	0.034257066	11.1761965	0.1297935	heart_17wk	heart	17wk	Medium-high
4.075580617	0.2458239	0.6059364	0.00080148	11.1761965	0.1297935	heart_18wk	heart	18wk	Medium-high
6.55960446	0.2458239	0.6059364	0.000262936	11.1761965	0.1297935	heart_20wk	heart	20wk	Medium-high
5.74418812	0.2458239	0.6059364	1.534169578	11.1761965	0.1297935	intestine_10wk	intestine	10wk	Medium-high
1.081610724	0.2458239	0.6059364	2.964718144	11.1761965	0.1297935	intestine_11wk	intestine	11wk	Medium-high
55.23371015	0.2458239	0.6059364	8.355695623	11.1761965	0.1297935	intestine_15wk	intestine	15wk	Medium-high
17.77655336	0.2458239	0.6059364	5.69073144	11.1761965	0.1297935	intestine_17wk	intestine	17wk	Medium-high
1.145043817	0.2458239	0.6059364	3.662984642	11.1761965	0.1297935	kidney_16wk	kidney	16wk	Medium-high
1.898032755	0.2458239	0.6059364	4.76650881	11.1761965	0.1297935	kidney_20wk	kidney	20wk	Medium-high
2.996985478	0.2458239	0.6059364	2.589069545	11.1761965	0.1297935	stomach_10wk	stomach	10wk	Medium-high
0.026501692	0.2458239	0.6059364	18.521657	11.1761965	0.1297935	stomach_16wk	stomach	16wk	Medium-high
0.497070901	0.2458239	0.6059364	2.417310572	11.1761965	0.1297935	heart_10wk	heart	10wk	Medium
0.298088224	0.2458239	0.6059364	1.915809404	11.1761965	0.1297935	kidney_10wk	kidney	10wk	Medium
0.251670635	0.2458239	0.6059364	8.631797944	11.1761965	0.1297935	lung_20wk	lung	20wk	Medium
0.055139948	0.2458239	0.6059364	1.422144401	11.1761965	0.1297935	lung_10wk	lung	10wk	Medium-low
0.230446752	0.2458239	0.6059364	5.9537349	11.1761965	0.1297935	lung_17wk	lung	17wk	Medium-low
0.052804662	0.2458239	0.6059364	10.46668006	11.1761965	0.1297935	stomach_20wk	stomach	20wk	Medium-low
0.144708839	0.2458239	0.6059364	0.007590666	11.1761965	0.1297935	adrenal_10wk	adrenal	10wk	low
0.04589555	0.2458239	0.6059364	0.000286753	11.1761965	0.1297935	adrenal_16wk	adrenal	16wk	low
0.178886465	0.2458239	0.6059364	0.01424403	11.1761965	0.1297935	adrenal_18wk	adrenal	18wk	low
0.172930422	0.2458239	0.6059364	0.032532795	11.1761965	0.1297935	adrenal_20wk	adrenal	20wk	low
